# Can facet joint block be a complementary or alternative therapeutic option for patients with osteoporotic vertebral fractures: a meta-analysis

**DOI:** 10.1186/s13018-022-02933-9

**Published:** 2022-01-21

**Authors:** Zhi Chen, Chenyang Song, Jianwen Chen, Jun Sun, Wenge Liu

**Affiliations:** 1 Department of Orthopedics Surgery, Fujian Medical University Union Hospital, Fuzhou, 350001 Fujian China; 2 Department of Orthopedics Surgery, The First People’s Hospital of Yulin, Yulin, 537000 Guangxi China; 3 Department of Emergency, Zhaotong Traditional Chinese Medicine Hospital, Zhaotong, 657000 Yunnan China

**Keywords:** Facet joint block, Vertebroplasty, Kyphoplasty, Osteoporotic vertebral compression fracture, Meta-analysis

## Abstract

**Background:**

Recently facet joint block has been increasingly used to relief the residual pain after vertebral augmentation, but whether it can be a complementary or alternative to vertebral augmentation remain largely unknown. Thus, we conducted this meta-analysis to determine the effect of facet joint block in the treatment of osteoporotic vertebral compression fractures (OVCF).

**Methods:**

Following PRISMA statement, a comprehensive literature search through Embase, PubMed, Web of Science, Wanfang Data, China National Knowledge Infrastructure and Chinese BioMedical Literature Database was performed to identify relevant studies. Studies comparing vertebral augmentation combined with facet joint block (combined therapy) with vertebral augmentation, and studies comparing facet joint block with vertebral augmentation were analyzed, respectively.

**Results:**

A total of 10 studies were included. There were seven studies comparing combined therapy with vertebral augmentation, the results showed combined therapy was associated with significantly lower visual analog scale (VAS) scores on postoperative day 1, 7, month 1, 3, and lower oswestry disability index (ODI) scores on postoperative day 1, 7, and month 3. There were three studies comparing facet joint block with vertebral augmentation, the results demonstrated vertebral augmentation only provided better analgesia in month 1 after surgery, but it was associated with a higher incidence of refracture.

**Conclusions:**

Current evidence suggested facet joint block might be considered as a complementary to vertebral augmentation in the treatment of OVCF, but it might not be effectively used as an alternative therapy.

**Supplementary Information:**

The online version contains supplementary material available at 10.1186/s13018-022-02933-9.

## Background

With the aging population, the prevalence of OVCF is gradually increasing. Vertebral augmentation, including percutaneous vertebroplasty (PVP) and percutaneous kyphoplasty (PKP), was once considered as a highly effective method in the treatment of OVCF. However, more recent studies demonstrated a significant number of patients suffered from residual pain and functional disability after surgery [1–3].

Currently, the underlying mechanism for the residual pain is yet to be established. Most of previous studies have focused on the fracture site, while the role of posterior elements is often disregarded [4, 5]. Until recently, the importance of posterior supporting structures has been gradually elucidated. Doi et al. found that the collapse of vertebral body could lead to additional load and stress in the posterior elements [4]. Lehman et al. observed abnormal facet joint signal changes in patients with acute or subacute OVCF [6]. Furthermore, Park et al. demonstrated facet joint block could provide significant pain relief for patients with OVCF complaining of residual pain after vertebral augmentation, which supported the hypothesis that facet joint might be another source of pain [3].

In light of these findings, several studies have been conducted to investigate the efficacy of facet joint block as a complementary or an alternative to vertebral augmentation, but the results remain controversial [7–10]. Therefore, we conducted this meta-analysis to determine the effect of facet joint block in OVCF treatment.

## Methods

### Search strategy

This meta-analysis was registered on the PROSPERO (CRD42021262828) and conducted following the Preferred Reporting Items for Systematic Reviews and Meta-Analyses (PRISMA) statement (Additional file 1: The PRISMA checklist) [11]. After consensus has been reached, two researchers independently performed a comprehensive literature search through Embase, PubMed, Web of Science (WOS), Wanfang Data, China National Knowledge Infrastructure (CNKI) and Chinese BioMedical Literature Database (CBM) on June 18, 2021 to identify relevant studies. The following combinations of search terms were used in the systematic search: (vertebral fracture OR spinal fracture OR spine fracture) AND (vertebroplasty OR kyphoplasty OR PVP OR PKP) AND (facet joint block OR facet joint injection OR zygapophysial joint block OR zygapophysial joint injection OR zygapophyseal joint block OR zygapophyseal joint injection OR medial branch block OR nerve block). The search was restricted to clinical trials published in Chinese and English, and the references of all included studies were also reviewed for potential eligible studies.

### Inclusion/exclusion criteria

Studies that met the following inclusion criteria were included: (1) Participants: patients with osteoporotic vertebral compression fractures; (2) Interventions: facet joint block combined with vertebral augmentation (combined therapy) or facet joint block alone; (3) Comparison: vertebral augmentation; (4) Outcomes: postoperative VAS scores, ODI scores and the incidence of refracture.

The exclusion criteria were as follows: (1) Secondary fractures caused by tumor, infection or bone metabolic diseases; (2) Studies comparing vertebral augmentation with other nerve block techniques (e.g., gray ramus communicans nerve block, nerve-root block or radiofrequency denervation); (3) Studies with insufficient data for calculating the results; (4) Conference abstracts, letters, case reports, reviews, duplicated studies and in vitro studies.

### Data extraction and quality assessment

Based on the inclusion and exclusion criteria, two researchers independently screened the searched studies and extracted data from the eligible studies. Any discrepancy was resolved by consulting with a third author. For each included study, the following data were collected: first author, published year, country, study design, age, gender, sample size, type of surgery, anesthetic agent, VAS scores at postoperative day 1 and 7, month 1, 3, 6 and 12, ODI scores at postoperative day 1 and 7, month 1, 3, 6 and 12, the incidence of refracture and length of follow-up. The quality of each randomized controlled trial (RCT) and cohort study was evaluated using Jadad scale [12] and Newcastle–Ottawa Scale (NOS) [13], respectively. It was considered as a high-quality study, when the Jadad score was greater than 3 or NOS score was greater than 6.

### Statistical analysis

This meta-analysis was conducted using STATA 12.0 software. We calculated weighted mean difference (WMD) with 95% confidence interval (CI) for continuous variables, and odds rations (OR) with 95% CI for dichotomous variables. A 2-sided *P* < 0.05 was considered statistically significant. Heterogeneity was evaluated using the I-squared (*I*^2^) test. Heterogeneity of effects was identified when *I*^2^ > 50%, and then the random effect model was applied and a sensitivity analysis was conducted. Otherwise, the fixed effect model was used.

## Results

### Search results

**Fig. 1:**
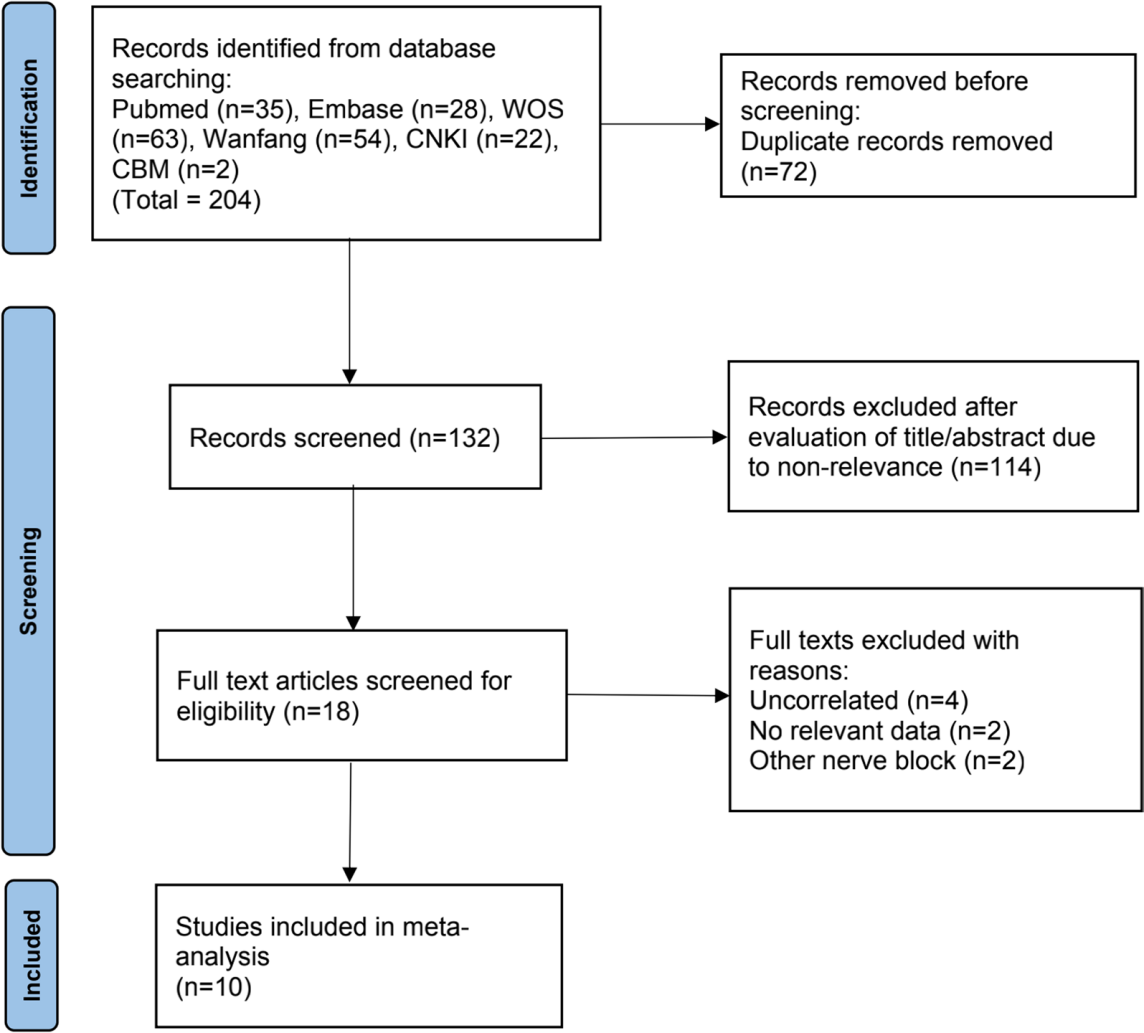
Flowchart of study selection

The flow chart of study selection processes is shown in Fig. 1. The initial search identified 204 relevant studies, and 132 studies were retained after removal of duplicates. Of these, 114 studies were eliminated after screening on titles and abstracts, another eight studies were excluded after full-text review. Finally, 10 eligible studies were included in this meta-analysis.

### Study characteristics and quality evaluation

The characteristics of included studies are presented in Table 1. There were seven studies comparing combined therapy with vertebral augmentation [7, 10, 14–18], and three studies comparing facet joint block with vertebral augmentation [8, 9, 19]. A total of 1145 patients were included, the mean age ranged from 62.59 to 85.7 years old and the follow-up period ranged from 1 to 24 months. In most studies lidocaine combined with steroid were used for facet joint block, but Tao et al. used lidocaine alone, Li et al. used ropivacaine combined with steroid and vitamin B12, and Cheng et al. used lidocaine combined with mecobalamin. According to the Jadad Scale and NOS, nine out of ten included studies were categorized as high-quality studies.

**Table 1:** The baseline characteristics of included studies

References	Year	Country	Study	Anesthetic agent	Treatment	Patients	Age	Sex(M/F)	Control	Patients	Age	Sex(M/F)	Follow up	Quality
Tao [18]	2020	China	Cohort study	Lidocaine	PKP + FJB	31	74.48	10/21	PKP	29	77.52	11/19	1 mon	H
Zhang [17]	2018	China	Cohort study	Lidocaine + steroid	PKP + FJB	56	NA	NA	PKP	56	NA	NA	3 mon	H
Li [16]	2016	China	Cohort study	Lidocaine + steroid	PVP + FJB	19	84.6	5/14	PVP	23	85.7	7/16	4–18 mon	H
Zhang [15]	2021	China	RCT	Lidocaine + steroid	PVP + FJB	34	78.32	19/14	PVP	34	78.65	20/14	6 mon	L
Wang [14]	2019	China	Cohort study	Lidocaine + steroid	PKP + FJB	36	76.14	15/21	PKP	36	75.86	14/22	1 mon	H
Li [10]	2021	China	Cohort study	Ropivacaine + steroid + vitamin B12	PKP + FJB	83	65.3	29/54	PKP	88	66.8	32/56	12 mon	H
Cheng [7]	2020	China	Cohort study	Lidocaine + steroid	PKP + FJB	79	72.9	12/67	PKP	125	70.8	25/100	12 mon	H
Luo [19]	2020	China	Cohort study	Lidocaine + mecobalamin	FJB	23	75.79	5/18	PKP	23	75.47	4/19	12 mon	H
Wang [8]	2016	China	RCT	Lidocaine + steroid	FJB	106	62.59	22/84	PVP	100	63.68	19/81	12 mon	H
Bae [9]	2018	USA	Cohort study	Lidocaine + steroid	FJB	72	73.1	19/53	PVP	92	76.7	24/68	24 mon	H

RCT, randomized controlled trial; PKP, percutaneous kyphoplasty; PVP, percutaneous vertebroplasty; FJB, facet joint block; NA, not available; Mon, month; H, high; L, low

### Clinical outcome analysis

In studies comparing combined therapy with vertebral augmentation, the pooled results showed that combined therapy was associated with significantly lower VAS scores on postoperative day 1 and 7, month 1 and 3, but there was no significant difference on postoperative month 6 and 12. Similarly, significantly improved ODI scores was observed in combined therapy on postoperative day 1, 7, month 3, but there was no significant difference on postoperative month 1, 6 and 12 (Table 2). The sensitivity analysis of VAS score at postoperative month 3 and ODI scores at postoperative day 1 and month 3 were demonstrated in Additional file 2: Figure S1, Additional file 3: Figure S2 and Additional file 4: Figure S3.

**Table 2:** The results of combined therapy versus vertebral augmentation

Variables	Number of studies	Model	WMD	95% CI		*I*^2^ (%)	*P*
*VAS*							
1 day	7	Fixed	− 1.48	− 1.565	− 1.39	0.482	**0.000**
7 day	2	Random	− 1	− 1.859	− 0.14	0.906	**0.022**
1 mon	3	Random	− 0.6	− 0.896	− 0.3	0.899	**0.000**
3 mon	5	Random	− 0.71	− 0.987	− 0.42	0.926	**0.000**
6 mon	2	Random	− 0.51	− 1.656	0.63	0.707	0.379
12 mon	2	Random	− 0.12	− 0.31	0.076	0.72	0.233
*ODI*							
1 day	6	Random	− 8.47	− 11.09	− 5.84	0.917	**0.000**
7 day	3	Random	− 9.97	− 19.45	− 0.49	0.995	**0.039**
1 mon	3	Random	− 4	− 10.23	2.228	0.98	0.208
3 mon	5	Random	− 5.96	− 9.122	− 2.8	0.953	**0.000**
6 mon	2	Random	− 3.1	− 10.52	4.312	0.885	0.412
12 mon	2	Random	− 0.06	− 0.832	0.72	0.566	0.888

VAS, Visual Analogue Scale; ODI, Oswestry Disability Index; Mon, month

**Fig. 2:**
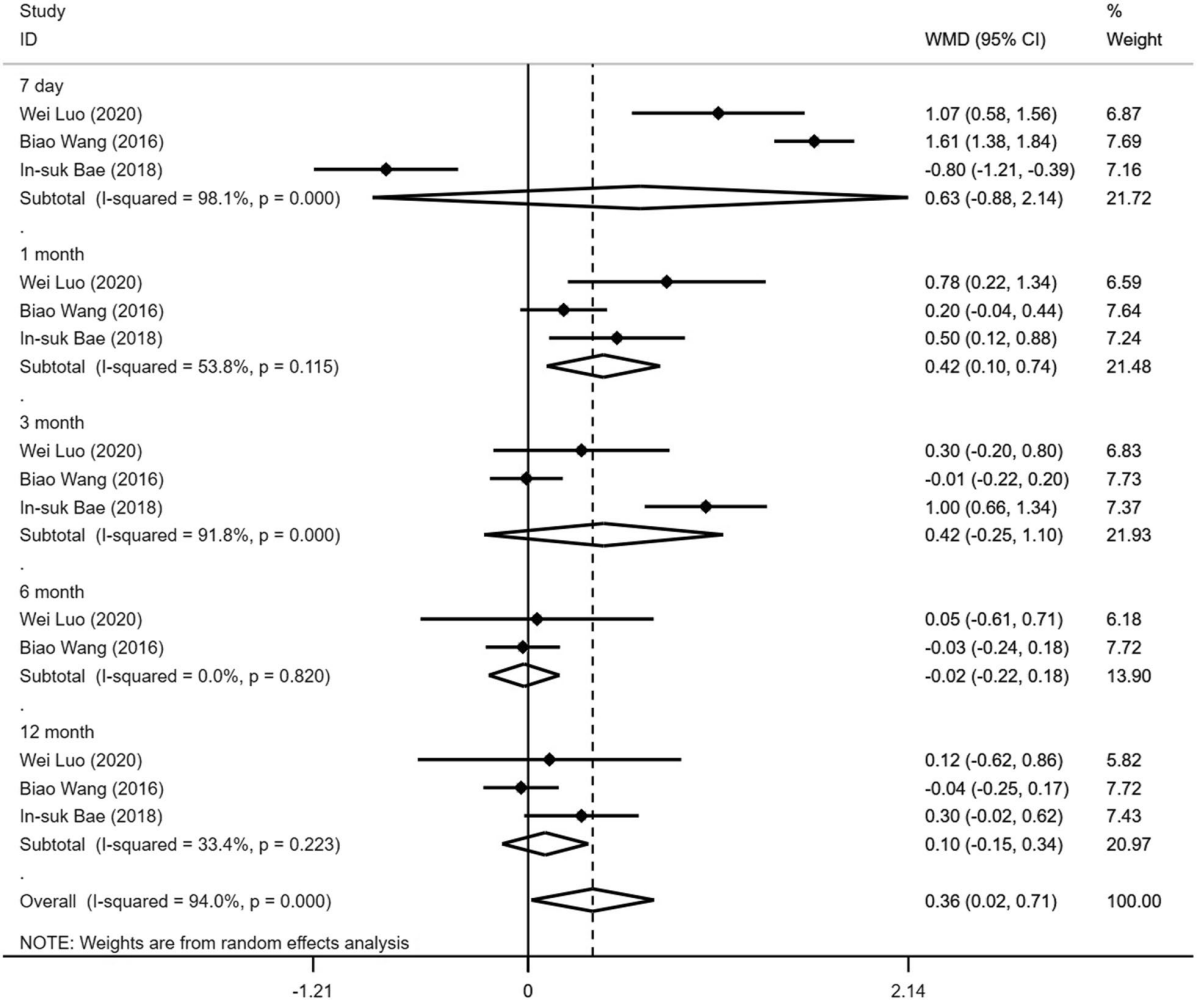
Forest plot of the postoperative VAS scores comparing facet joint block with vertebral augmentation

**Fig. 3:**
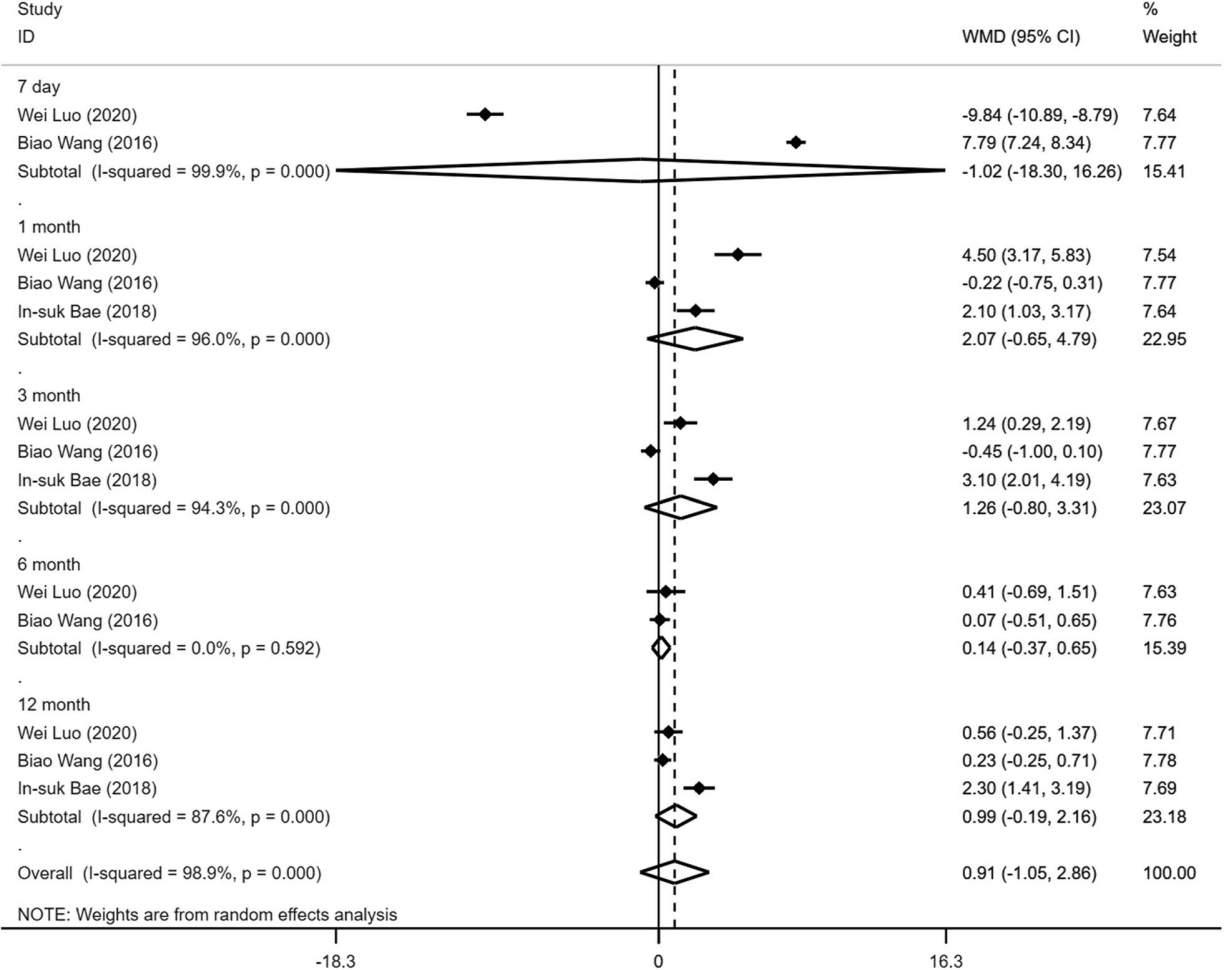
Forest plot of the postoperative ODI scores comparing facet joint block with vertebral augmentation

Regarding studies comparing facet joint block with vertebral augmentation, the pooled results demonstrated a significantly higher VAS score on postoperative month 1 in facet joint block, but there was no significant difference on postoperative day 7, month 3, 6 and 12 (Fig. 2; Table 3). In addition, there was no significant difference in postoperative ODI scores between the two groups during the follow-up (Fig. 3; Table 3).

**Table 3:** The results of facet joint block versus vertebral augmentation

Variables	Number of studies	Model	WMD	95% CI		*I*^2^ (%)	*P*
*VAS*							
7 day	3	Randome	0.631	− 0.876	2.138	0.981	0.412
1 mon	3	Randome	0.423	0.104	0.743	0.538	**0.009**
3 mon	3	Randome	0.425	− 0.248	1.098	0.918	0.215
6 mon	2	Randome	− 0.023	− 0.222	0.177	0	0.824
12 mon	3	Randome	0.066	− 0.106	0.238	0.334	0.454
*ODI*							
7 day	2	Randome	− 1.019	− 18.3	16.258	0.999	0.908
1 mon	3	Randome	2.073	− 0.648	4.795	0.96	0.135
3 mon	3	Randome	1.255	− 0.803	3.313	0.943	0.232
6 mon	2	Randome	0.143	− 0.368	0.654	0	0.583
12 mon	3	Randome	0.988	− 0.186	2.162	0.876	0.099

VAS: Visual Analogue Scale, ODI: Oswestry Disability Index, Mon: month

**Fig. 4:**
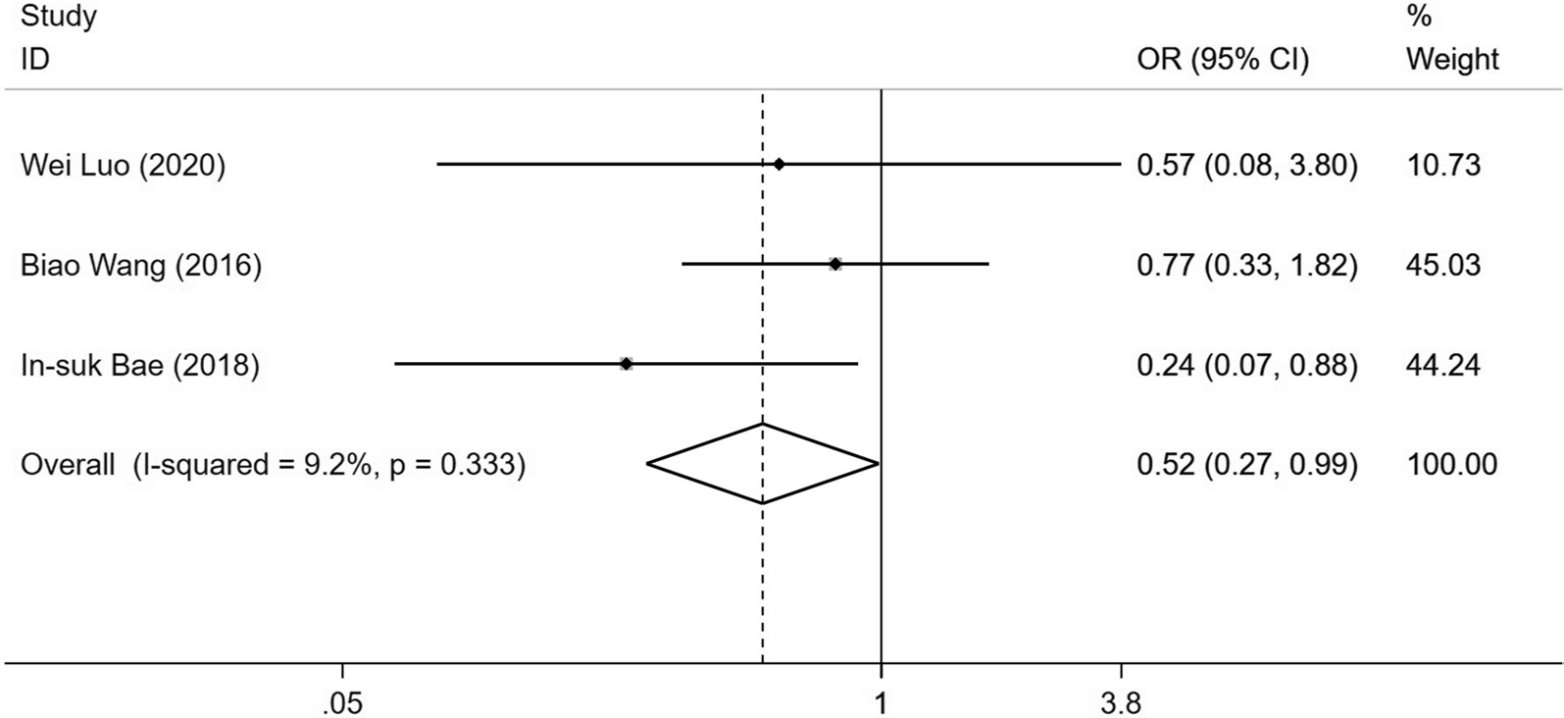
Forest plot of the incidence of refracture comparing facet joint block with vertebral augmentation

As for the incidence of refracture, the pooled result showed a significantly lower incidence of refracture in the facet joint block (OR 0.517; 95% CI0.271–0.986; fixed effect model; *I*^2^ 9.2%) (Fig. 4).

## Discussion

Although vertebral augmentation is widely performed in the treatment of OVCF, it is not universally successful. Based on previous literatures, approximately 14–23.6% of patients suffered from persistent or recurrent pain after vertebral augmentation [10, 20]. Worse yet, additional interventional procedures were required in about 21–24% of these patients for pain relief, which brought huge burden on patients and society [20, 21]. To the best of our knowledge, this is the first meta-analysis investigating the effect of facet joint block to the treatment of OVCF. Our findings suggested the combination of facet joint block and vertebral augmentation could improve the clinical outcomes of patients suffering from OVCF.

Recently the residual pain after vertebral augmentation has attracted the attention of spine surgeons. Some scholars hold the view that residual pain might not be due to a failed operation, but rather to a untreated or a new pain generator [22]. Biomechanical studies showed that secondary kyphotic deformity caused by vertebral fracture could induce subluxation of the posterior structures and increase local stresses, which might be another pain generator [5, 23]. There was no doubt that pain originating from the posterior structures was unlikely to benefit from vertebral augmentation [5]. Thus, some researchers hypothesized the additional treatment of posterior elements might improve the clinical outcomes of patients with OVCF. In a retrospective study, Wang et al. found significantly lower VAS and ODI scores in the combined therapy group, but this was only observed up to 72 h postoperatively [14]. In another prospective randomized study, Li et al. reported the additional facet joint block could significantly improve the VAS and ODI scores within the first month after the operation [10]. However, there were some slight differences in Chen et al.’s study, the results showed the combined therapy could only accelerate the pain relief, the ODI scores did not differ significantly between the two groups [7]. The pooled results of our meta-analysis confirmed that the combined therapy could not only facilitate pain relief within the first 3 months, but also accelerate function recovery up to 7 days postoperatively. Our finding was consistent with a previous study, the anesthetic agents used in the facet joint block could provide approximately 13.26 weeks of pain relief [3]. Thus, the combined therapy is recommended for patients with risk factors associated with posterior pain, such as severe kyphotic deformity and abnormal facet joint signals [6, 24].

Although facet joint block appears to be a useful adjuvant to the treatment of OVCF, it remains controversial whether it can replace vertebral augmentation as a better choice. Wilson et al. indicated only a third of acute OVCF patients technically suitable for PVP could be successfully treated with facet joint block [25]. Im et al. observed approximately half of patients experienced continued pain after facet blocking prior to PVP [24]. In a prospective randomized controlled trial including 206 patients, Wang et al. reported a greater improvement of VAS and ODI scores for patients in the PVP group than those in the facet blocking group [8]. In another study published in Chinese, the authors also found superior analgesic effect and functional recovery in patients receiving PKP at postoperative week 1 and month 1, when comparing with those receiving facet joint block [19]. On the contrary, Bae et al. demonstrated that both PVP and facet joint block provided significant pain relief for patients with single-level OVCF [9].Our meta-analysis clarified that vertebral augmentation provided better pain relief only at postoperative month 1, and the difference in the improvement of ODI scores between the two procedures was insignificant.

In the literature, the incidence of refracture after vertebral augmentation ranged from 2 to 52% [26, 27], which was a serious complication that urgently needs to be solved. Although several studies have been conducted to find an effective alternative, the results were debatable. While Luo et al. reported no significant difference in the occurrence of refracture between facet blocking and PKP groups [19]. Wang et al. found a slightly higher rate of refracture in the PVP group [8]. In another study, Bae et al. showed that there was a significantly higher incidence of refracture in the PVP group compared with facet blocking group [9]. Based on the limited published studies, our meta-analysis indicated that vertebral augmentation was associated with higher refracture risk comparing with facet joint block. It is likely that cement augmentation increases the strength and stiffness of the fractured vertebrae, resulting in greater stress on the adjacent vertebrae and subsequently higher risk of refracture [28].

### Limitation

There were some limitations in our study. Firstly, the number of eligible studies was limited and most studies were retrospective in nature. Secondly, the sample size was relatively small, which prevented us from drawing a stronger conclusion. Thirdly, the anesthetic agents and cement augmentation techniques in the various studies were not completely consistent, which might influence the outcomes.

## Conclusion

Current evidence suggested that facet joint block might be considered as a complementary to vertebral augmentation in the treatment of OVCF, especially for patients with risk factors associated with posterior pain. However, the facet joint block alone could not be used as an effective alternative to vertebral augmentation.

## Supplementary Information

**Additional file 1.** The PRISMA checklist.

**Additional file 2: Figure S1.** Sensitivity analysis of VAS score at postoperative month 3.

**Additional file 3: Figure S2.** Sensitivity analysis of ODI score at postoperative day 1.

**Additional file 4: Figure S3.** Sensitivity analysis of ODI score at postoperative month 3.

## Data Availability

The data are available from the corresponding author on reasonable request.

## Abbreviations

OVCF: Osteoporotic vertebral compression fractures
PVP: Percutaneous vertebroplasty
PKP: Percutaneous kyphoplasty
PRISMA: Preferred reporting items for systematic reviews and meta-analyses
WOS: Web of science
CNKI: China National Knowledge Infrastructure
CBM: Chinese BioMedical Literature Database
VAS: Visual Analogue Scale
ODI: Oswestry Disability Index
RCT: Randomized controlled trial
NOS: Newcastle–Ottawa Scale
