# Supplementary figures and images for: Can facet joint block be a complementary or alternative therapeutic option for patients with osteoporotic vertebral fractures: a meta-analysis
Source: J Orthop Surg Res. 2022 Jan 21;17:40. doi: 10.1186/s13018-022-02933-9 (PMC8781236; doi:10.1186/s13018-022-02933-9)

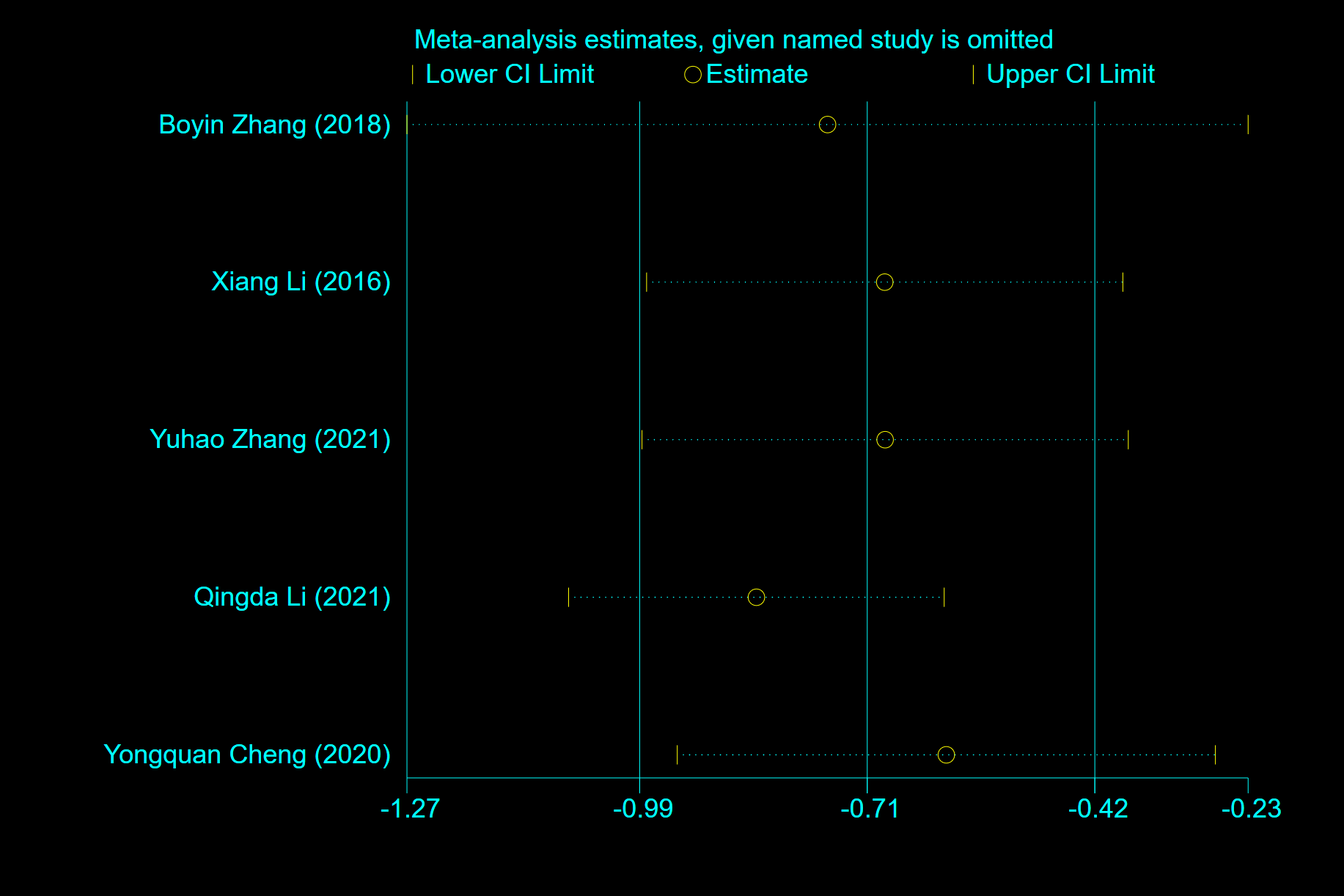

Supplement: Supplementary file 2 — Additional file 2: Figure S1. Sensitivity analysis of VAS score at postoperative month 3. [file 13018_2022_2933_MOESM2_ESM.tif]

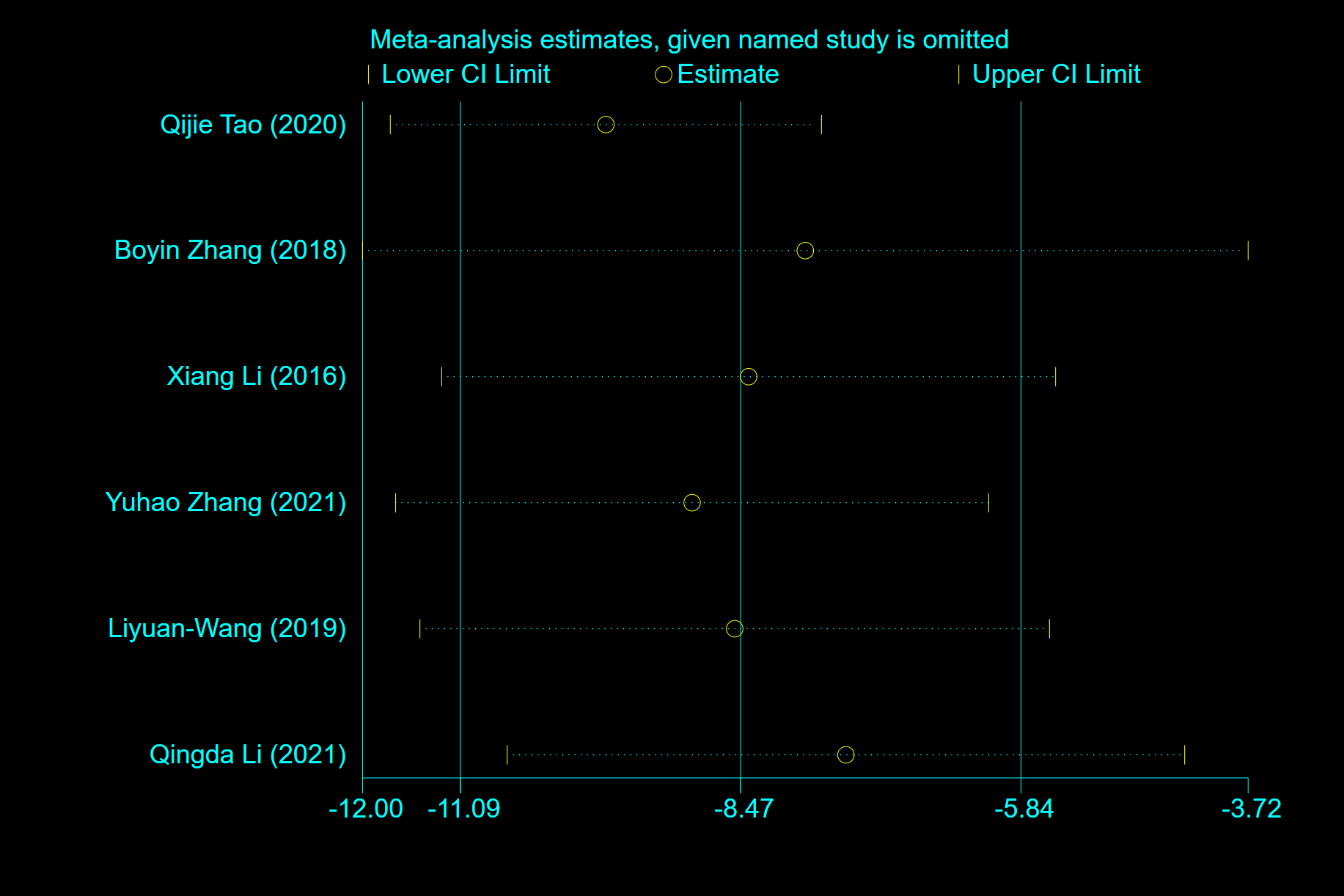

Supplement: Supplementary file 3 — Additional file 3: Figure S2. Sensitivity analysis of ODI score at postoperative day 1. [file 13018_2022_2933_MOESM3_ESM.tif]

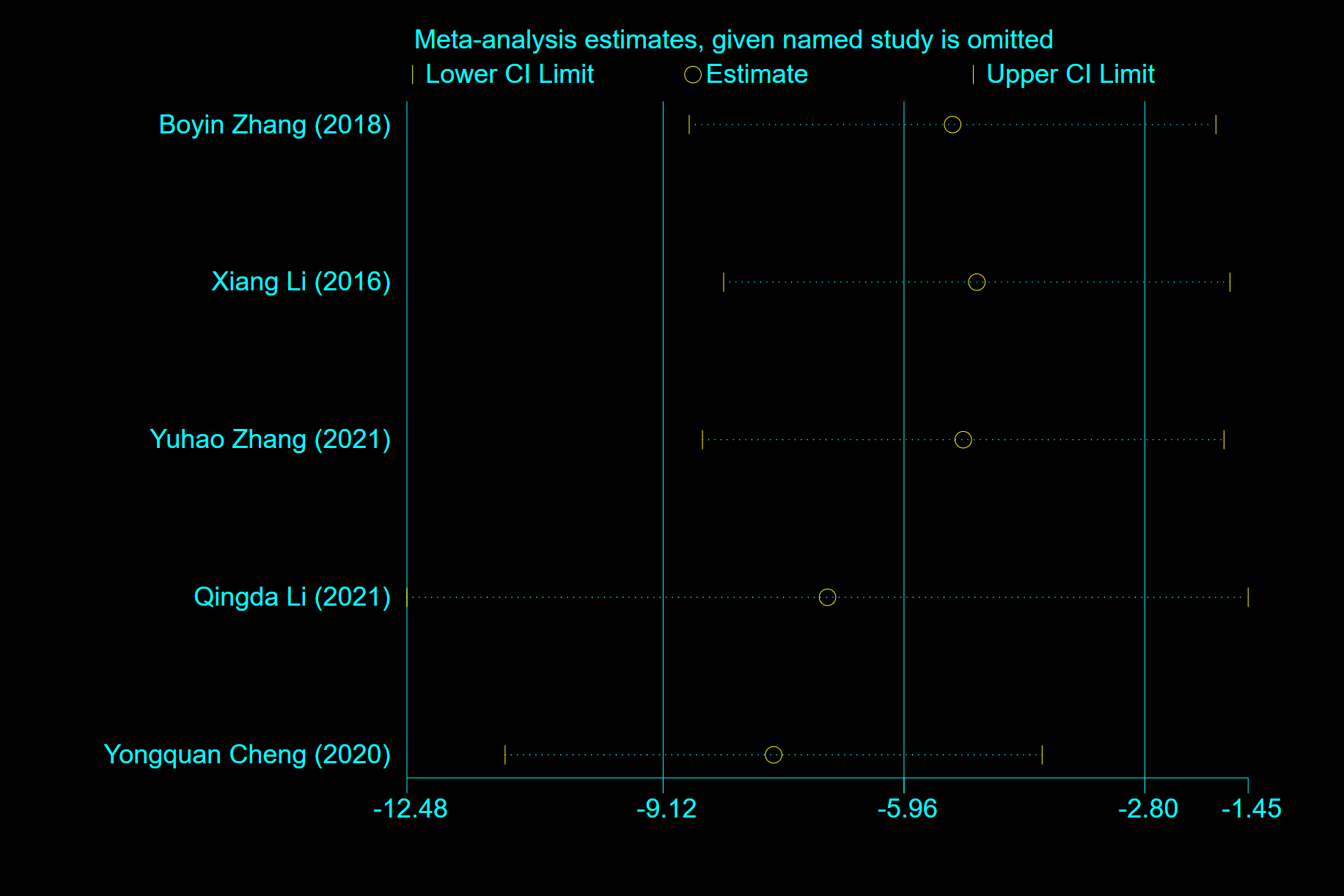

Supplement: Supplementary file 4 — Additional file 4: Figure S3. Sensitivity analysis of ODI score at postoperative month 3. [file 13018_2022_2933_MOESM4_ESM.tif]
